# Positron emission tomography-based assessment of metabolic gradient and other prognostic features in sarcoma

**DOI:** 10.1117/1.JMI.5.2.024502

**Published:** 2018-05-24

**Authors:** Eric Wolsztynski, Finbarr O’Sullivan, Eimear Keyes, Janet O’Sullivan, Janet F. Eary

**Affiliations:** a University College Cork, Statistics Department, Cork, Ireland; b National Cancer Institute, Bethesda, Maryland, United States

**Keywords:** FDG-positron emission tomography, heterogeneity, metabolic gradient, spatial modeling, texture, radiomics, prognosis, sarcoma, machine learning

## Abstract

Intratumoral heterogeneity biomarkers derived from positron emission tomography (PET) imaging with fluorodeoxyglucose (FDG) are of interest for a number of cancers, including sarcoma. A range of radiomic texture variables, adapted from general methodologies for image analysis, has shown promise in the setting. In the context of sarcoma, our group introduced an alternative model-based approach to the measurement of heterogeneity. In this approach, the heterogeneity of a tumor is characterized by the extent to which the 3-D FDG uptake pattern deviates from a simple elliptically contoured structure. By using a nonparametric analysis of the uptake profile obtained from this spatial model, a variable assessing the metabolic gradient of the tumor is developed. The work explores the prognostic potential of this new variable in the context of FDG-PET imaging of sarcoma. A mature clinical series involving 197 patients, 88 of whom have complete time-to-death information, is used. Texture variables based on the imaging data are also evaluated in this series and a range of appropriate machine learning methodologies are then used to explore the complementary prognostic roles for structure and texture variables. We conclude that both texture-based and model-based variables can be combined to achieve enhanced prognostic assessments of outcome for patients with sarcoma based on FDG-PET imaging information.

## 1 Introduction

The importance of quantitative assessment beyond semiquantitative SUV-based summaries is now firmly established in a number of contexts (diseases and modalities). Positron emission tomography (PET) has been found useful in the evaluation of intratumoral heterogeneity at the macroscopic level^1^ and calls for more elaborate algorithmic methodologies to capture prognostic information. This assessment can be achieved using spatial mathematical modeling of the metabolic tracer uptake information observed within the volume of interest (VoI). Our group has considered modeling the 3-D tumor uptake using a geometric (ellipsoidal) pattern of reference.^2^ With this approach, heterogeneity is defined as a lack-of-fit of the data to that idealized pattern. The ellipsoidal structural model provides an opportunity to construct further descriptors of both spatial and metabolic characteristics of the tumor.^3^ Such model-based assessment of the uptake distribution provides potential for further tumor characterization. Here, we develop an approach, involving a nonparametric analysis of the 3-D elliptical contour profile, for evaluating the “metabolic gradient” of the tumor at each voxel. These model-based volumetric gradients can be combined with structural heterogeneity in multivariate prognostic analyses. This methodology allows one to generate a number of related meaningful metabolic descriptors that may be considered independently or together, as they exhibit limited correlation with each other. Section 4 illustrates the prognostic value of these model-based characteristics, and in particular that of metabolic gradient assessment, which constitutes the main contribution of this paper.

This PET-based heterogeneity assessment can also be considered within the context of a larger set of radiomic features that may have prognostic value for patient outcome. Accounts of radiomics-based prognostic analyses using PET have been reported in a number of contexts, including for lung, oesophageal, breast, and head and neck cancers.^4^^–^^11^ Modern radiomic analysis considers general, and not necessarily biologically motivated, statistical characteristics of the distribution of tracer uptake. In most cases, they consist of first-order summaries of the uptake histogram and second-order texture features^12^ derived from relationships between neighboring voxels in the region under study. Texture analysis has also been used with CT (e.g., in lung^1^^,^^6^^,^^13^) and MRI data (e.g., in glioma^14^^,^^15^). Texture analyses for prognostic or therapeutic assessment of sarcoma are reported for CT or MRI data.^16^^–^^19^ A recent contribution by Vallières et al.^20^ has reported on the joint use of texture analysis with PET and MRI for prediction of lung metastases associated with primary sarcomas. In Sec. 3, we also explore the position of the proposed structural variables with respect to radiomic features, postulating that these two methodologies may be complementary to each other. In doing so, this paper illustrates that texture analysis offers opportunities for PET-based prognosis in sarcoma, as a secondary contribution.

The diversification of image-based quantitative metabolic assessments also naturally raises the question of feature selection for multivariate prognostic models, not unlike in genomics. Classical statistical solutions to this end can often meet their limitations since in many settings, the number p of covariates available could become greater than the number N of patients in the cohort. Stepwise selection for Cox hazard models may, for example, become unreliable. In this context, machine learning techniques can provide more adequate solutions toward feature selection. Such techniques are being considered more consistently in recent years^10^^,^^14^^,^^21^ for texture-based heterogeneity assessment. In Sec. 4, we also illustrate how final prognostic model selection may fair out when both sets of structural and textural features are considered together.

## 2 Methodology

### 2.1 Structural Modeling and Metabolic Gradients

Here, we describe the algorithmic approach used to construct a semiparametric regression profile of the volumetric uptake data in terms of a reference ellipsoidal pattern. Hereafter, the N observations (x,Y) are described by their 3-D coordinates x∈Ω in the image domain $\Omega \subset R^{3}$ and measured uptake value Y. The spatial distribution of uptake observations is characterized in terms of its compliance to a rigid 3-D ellipsoidal model parametrized by θ=(μ,Σ) for shape Σ (the uptake data covariance matrix) and location μ. Voxel location within this model can be expressed in terms of their radial position within the ellipsoid:

$$u(\theta )={\left(x-\mu \right)}^{T}\Sigma^{-1}(x-\mu ).$$ (1)

**Fig. 1:**
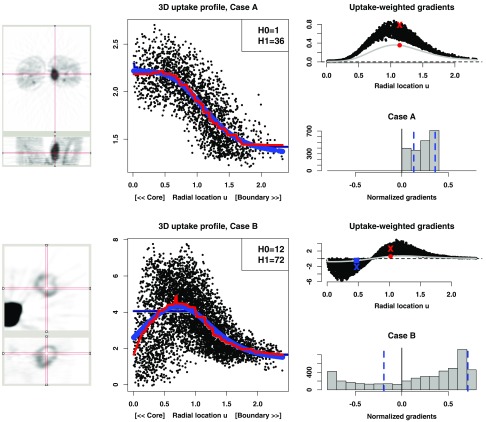
FDG-PET uptake profiles of two sarcoma studies: case A (49-year-old male with upper thigh soft tissue sarcoma, alive at last follow-up 4 years after baseline scan) presented with an active, homogeneous core (top) and case B (48-year-old male with pelvis soft tissue sarcoma, died 8 months after baseline scan) presented with a heterogeneous core with low activity (bottom). These features are visible from the transverse and coronal field-of-view images (left). Centre plots: corresponding uptake profile [u(θ^),Y], initial stepwise monotonically decreasing fit f[u(θ^)] (inner navy curve), stepwise unimodal regression f^[u(θ^)] (thinner, red curve), and smoothed bitonic uptake profile curve f˜[u(θ^)] (thicker, blue curve). Initial stepwise fit f(u) is barely noticeable for case A but highlights the benefit of using a unimodal fit in case B. Rightmost plots summarize gradient information: top plot illustrates the gradient curve g(u) Eq. (4), and uptake weighted gradients $g_{Y}(u)$, as functions of radial location u. Reference voxels are identified to illustrate decreasing and increasing uptake rates (a blue square and a red circle, respectively). The gradient lengths at those locations correspond to the absolute value of the normalized gradients $\tilde{g}$ Eq. (6). The plot for case B shows how the gradient measure has a negative sign for voxels located on the left hand side of the uptake profile mode (i.e., voxels closer to the core with a decreasing rate of uptake). Bottom plot: histogram of normalized gradients $\overset{˜}{g}$ on a common binning scale. Broken vertical lines indicate the 25th and 95th quantiles of normalized gradients.

This provides the opportunity to represent the volumetric uptake information as a function of ellipsoidal radius, as shown in the profile plot in Fig. 1. A nonparametric (either isotonic or bitonic) regression f of this profile (u,Y) can be obtained given a set choice of ellipsoidal parameters θ in the form of a model:

$$Y_{i}=f[u_{i}(\theta )]+\epsilon_{i},\;i=1,\ldots ,N,$$ (2)

where ${\left\{\epsilon_{i}\right\}}_{i=1}^{N}$ are realizations of a white noise process, assumed to have zero mean and constant variance. Our semiparametric approach thus consists in optimizing Eq. (2) over both the ellipsoidal shape and location θ and nonparametric regression curve f. In other words, the best nonparametric regression curve (in the least squares sense) is obtained for the optimal choice for θ, i.e., with respect to the ellipsoid that best fits the VoI uptake data.

The original approach^2^ consisted in fitting a stepwise isotonic decreasing nonparametric least squares regression function f to the uptake data Y, following the idealized representation of gradually decreasing ellipsoidal level curves at increasing ellipsoidal radii. A measure of lack-of-fit of this template structure was used directly to quantify heterogeneity (this is described in more details further). Here, we used this approach to quantify heterogeneity but introduced a second, bitonic (i.e., unimodal) regression for f in order to perform further assessment of the structure of the 3-D uptake distribution. This unimodal level profile f provides a more reasonable description of a tumor that might have a central necrotic core (see Fig. 1). (This principle also applies to other tumor types for which distributions have an overall decreasing radial uptake pattern, as it has the ability to differentiate sharper concentrations of uptake.) An algorithm to implement the unimodal fit is described in Appendix A. Given the fitted nonparametric unimodal uptake profile, it is now possible to evaluate associated gradients $-f^{\prime }[u(\theta )]$ at any point within the tumor. These signed uptake curve gradients can be used as a variable for tumor characterization (and prognostic assessment, as discussed hereafter). As the unimodal regression curve f^ obtained for Eq. (2) is a stepwise function, a smoothing spline is subsequently applied to it in order to obtain a final unimodal continuous uptake profile function $\overset{˜}{f}$, i.e.,

Y^i=f˜[ui(θ^)],i=1,…,N. (3)

This creates the opportunity to define a variable for tumor characterization in the form of the signed metabolic gradient:

g[u(θ^)]=−f˜′[u(θ^)], (4)

which yields a negative gradient value for a locally decreasing uptake profile (or locally decreasing metabolic activity) and a positive value at an area of increasing uptake signature (or increasing metabolic activity), as illustrated in Fig. 1. Here, “decreasing” is understood in terms of the tumor timescale, relative to peak activity and when exploring the VoI from the tumor core out toward the tumor boundary: for example, a more developed sarcoma will typically exhibit increased avidity further away from its core, and hence, core metabolic activity would be seen to decrease, as shown in Fig. 1. A technical description of this algorithm is provided in Appendix A.

### 2.2 Structural Variables and Interpretation

A number of structural variables can be extracted from the modeling approach described in Sec. 2.1 for tumor characterization. As in previous works from the group,^2^^,^^3^ intratumoral heterogeneity may be defined as a measure of lack-of-fit of the spatial model to the observed uptake distribution. Two versions of such a heterogeneity variable can be constructed as follows using Eq. (3):

H0=∑i=1N(Yi−Y^i)2∑i=1NYi2/N,H1=∑i=1N(Yi−Y^i)2Var(Y). (5)

Various gradient summaries may be obtained from Eq. (4) to further describe intratumoral status and activity. Normalized gradients may be derived from Eq. (4) to conform to a universal scale by

g˜[u(θ^)]=g[u(θ^)]max[(Y^)i=1N]. (6)

As each voxel i receives a gradient value gi=g[ui(θ^)], the sample of N signed gradients may be analyzed, for example, in terms of its first quartile or its 95th percentile, to capture summarizing features of the rate of metabolic change within the VoI. In this view, taking the first quartile of (normalized) gradients provides a single-valued evaluation at the lower end of the scale of metabolic rates: one would expect a lower first quartile for a tumor with decreasing metabolic activity, for example, resulting from necrosis. Likewise, the 95th percentile of (normalized) gradients provides an analysis of higher rates, where a higher value would likely correspond to a more rapidly changing tumor metabolism. Figure 1 illustrates this idea.

The sample of metabolic gradients may also be weighted by voxel uptake into

gY[u(θ^)]=Yg˜[u(θ^)] (7)

in order to emphasize evaluation at areas of higher uptake, before being summarized into quantiles of the vector (gY,i)i=1N={gY[ui(θ^)]}i=1N. The rightmost plot of Fig. 1 shows an example of such a sample.

### 2.3 Textural Quantitation

First-order, second-order, and regional features commonly found in other contributions^1^^,^^6^^,^^22^^–^^24^ were computed as per definitions provided by the Image Biomarker Standardization Initiative^25^ (version 1.5). Second-order features are defined as moments and functions of the grayscale level co-occurrence matrix (GLCM), which is made of the frequencies $P_{ij}$ of adjacency of any two uptake values (i,j) in a given direction.^12^ The output texture features are computed and averaged over all 13 directions in the volume. We use the normalized GLCM, i.e., $\sum_{i,j}P_{ij}=1$, where matrix P is symmetric. Regional features were derived from a gray level size zone matrix (GLSZM), which evaluates the numbers and sizes of contiguous homogeneous regions of equal (discretized) gray level.^22^^,^^24^ Abundant descriptions of the above-mentioned features can be found in the literature cited above. The list of features considered here is by no means comprehensive but includes the main first- and second-order features found in the literature on PET-derived radiomics. The two regional features were selected on the basis of their relevance in other works,^6^^,^^22^^,^^24^ their relative algorithmic simplicity, and to introduce higher-order evaluations in our analysis.

Other variables commonly found in the literature in the context of radiomics include assessment of shape^26^ or morphology.^24^^,^^25^ We also included in our analyses an evaluation of volume asphericity^26^ proportionally to the ratio of the segmented boundary surface S and its volume V by $S/{\left(36V\pi \right)}^{1/3}-1$. A comparison of morphological ellipsoidal features^25^ with our model-derived features was aptly suggested by a reviewer. On this basis, five morphological descriptors for ellipsoidal characteristics^25^ were included that rely on volume eigenvalues $\lambda_{\text{major}}\geq \lambda_{\text{minor}}\geq \lambda_{\text{least}}$ derived by principal component analysis (PCA). These features assess major, minor, and least axis lengths (defined, respectively, as $4\sqrt{\lambda_{\text{major}}},4\sqrt{\lambda_{\text{minor}}}$, and $4\sqrt{\lambda_{\text{least}}}$) as well as volume elongation and flatness ($\sqrt{\lambda_{\text{minor}}/\lambda_{\text{major}}}$ and $\sqrt{\lambda_{\text{least}}/\lambda_{\text{major}}}$, respectively).

## 3 Experimental Methods

### 3.1 Dataset and Analysis Framework

The dataset of primary sarcoma tumors was acquired at the University of Washington in Seattle, United States, between August 1993 and January 2003, after patients were diagnosed by biopsy. After exclusion of unsuitable cases due to lack of complete data availability, the cohort (N=197, 88 deaths observed) is composed of 130 soft tissue, 51 bone, and 16 cartilage sarcomas, in patients aged between 17 and 86 years of age (median 45), of which 86 females and 111 males, with 99 high-grade, 66 intermediate, and 32 low-grade tumors.

All patients underwent standard [18F] FDG-PET on a GE Advance PET scanner before either neoadjuvant chemotherapy or surgical resection. Patients received an intravenous injection of [18F] FDG (259-370 MBq) after fasting for at least 12 h and rested for between 45 and 60 min before emission and attenuation scan acquisition of the tumor fields of view (FoVs). All imaging data were reconstructed using filtered backprojection (FBP), rendering 3-D images using a Hanning filter after scatter correction, resulting in a reconstructed resolution of 10 mm.^27^ Output image sets had voxel size of 4.30  mm×4.30  mm in the transverse plane and slice thicknesses of 4.25 mm. Details on the imaging protocol were reported in previous reports.^2^^,^^28^ Raw counts were scaled into SUV with respect to activity in injected dose per unit weight of the patient (kBq/g).

Primary tumors were identified by a radiologist. Crude input ellipsoidal VoIs were drawn around the entire FDG-PET tumor volume in AMIDE^29^ for volumetric (3-D) statistical analysis. Our model-based quantitation approach is robust to the choice of input VoI, and in fact, the analysis can be performed on the crude bounding box (or bounding ellipsoid),^2^^,^^3^ which could include a (relatively small) proportion of background voxels. However, texture features do require volume segmentation, which was performed in three different ways in our initial analyses. Here, we present results obtained for a fixed-threshold segmentation similarly, e.g., to Yan et al.,^30^ where the threshold value is set for each study based on the subsample of the lower 15% of uptake values (so as to include background and healthy tissue activity only). For a given study, the segmentation threshold is defined as the mean subsample value plus three standard deviations of this subsample. This approach yielded satisfactory delineation throughout the cohort. Alternative segmentation techniques considered consisted in using either the convex hull of this threshold-segmented volume or the volume segmented obtained by localized fitting of a tubular representation.^3^ Results for these alternative approaches are left out here, as the analysis of their impact is outside the scope of this paper and may be considered in future works. The general conclusions of this paper remained unchanged by the choice of segmentation strategy.

Given the near-homogeneous voxel dimensions, no interpolation was performed prior to VoI resegmentation for texture analyses. Thus, both structural and textural groups of features were obtained from the same segmented VoI. Uptake values Y were requantized into Q=32 gray levels $Y^{Q}$ by fixed bin number transformation:

$$Y^{Q}=\lfloor \frac{\left(Q-1)[Y-\min(Y)\right]}{\max(Y)-\min(Y)}\rfloor +1.$$

Experimentation indicated that although texture quantitation changed with the choice of Q, this did not meaningfully impact general conclusions from our survival analyses. We have not considered the impact of alternative quantization techniques; this question is beyond the scope of this paper.

A total of 44 variables were considered and may be identified in three frames as follows: (i) routine clinical variables [tumor grade, clinical volume, clinical tumor subtype, patient age, patient sex, maximum standardized uptake value (SUVmax), mean uptake value (SUVmean) and total lesion glycolysis (TLG) were collected for this cohort]; (ii) structural features including heterogeneity using both $H_{0}$ and $H_{1}$, and the raw, normalized, and uptake-weighted gradients (Sec. 2.2); and (iii) a set of image summaries including morphologic and texture features (Sec. 2.3).

The primary endpoint for all survival analyses was overall survival status at last follow-up. The sarcoma cohort comprises tumors of three different grades and subtypes described above. In particular, it includes a high number of high grade soft tissue sarcomas (STS). For this reason, we excluded a total of 15 patients, who were lost to follow-up within the first 2 years for survival analyses, a common horizon in survival studies of STS.^31^ This threshold seems a reasonable compromise as median survival times can drop to 11 months for higher stages, irrespective of grade, according to the TNM classification of malignant tumours (TNM).^31^^,^^32^

Univariate Cox survival analyses performed for all of the variables revealed the following were significant, at least at the 5% significance level: (clinical) tumor grade, subtype, tumor volume, SUVmax, SUVmean, and TLG; (structural) $H_{0}$, $H_{1}$, ${\overset{˜}{g}}_{\left[0.25\right]}$, $g_{Y,[0.95]}$; (textural) asphericity, ${\mathrm{PCA}}_{\mathrm{major}}$, ${\mathrm{PCA}}_{\mathrm{minor}}$, ${\mathrm{PCA}}_{\mathrm{least}}$, ${\mathrm{mean}}_{\mathrm{HIST}}$, ${\text{skewness}}_{\mathrm{HIST}}$, ${\text{kurtosis}}_{\mathrm{HIST}}$, ${\text{median}}_{\mathrm{HIST}}$, ${\text{entropy}}_{\mathrm{HIST}}$, ${\text{uniformity}}_{\mathrm{HIST}}$, $\min.{\mathrm{gradient}}_{\mathrm{HIST}}$, ${\text{entropy}}_{\mathrm{GLCM}}$, ${\text{dissimilarity}}_{\mathrm{GLCM}}$, ${\text{homogeneity}}_{\mathrm{GLCM}}$, ${\text{contrast}}_{\mathrm{GLCM}}$, ${\text{autocorrelation}}_{\mathrm{GLCM}}$, and ${\max.\mathrm{probability}}_{\mathrm{GLCM}}$.

### 3.2 Feature Space Exploration

#### 3.2.1 Correlation

**Fig. 2:**
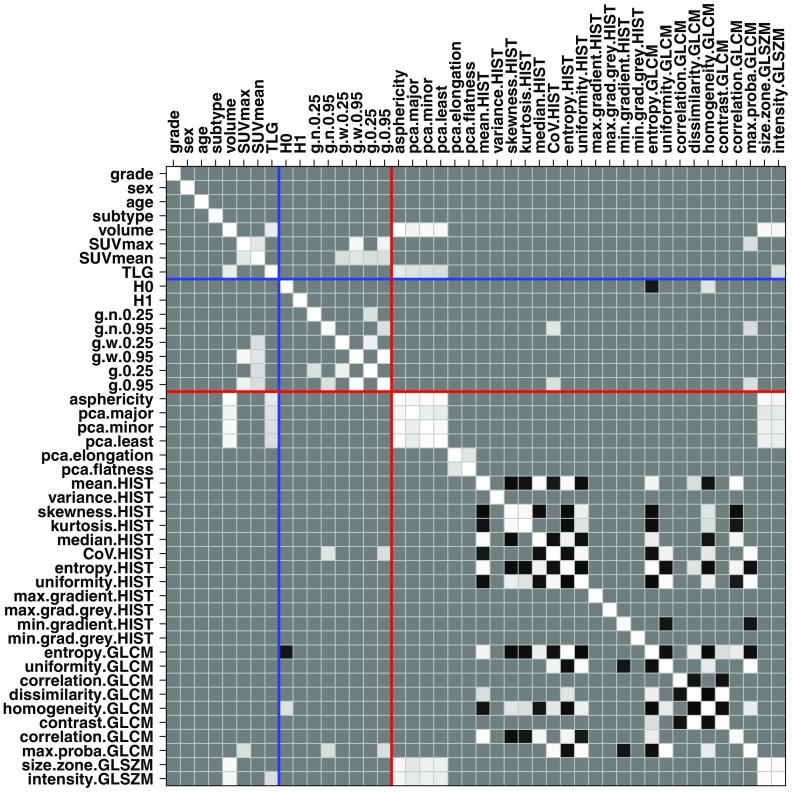
Correlation matrix of the continuous variables used in the survival analyses. All correlations within (−0.65,+0.65) have been reduced to the predominant gray level in the image. Gray levels darker and brighter than that level indicates, respectively, negative and positive correlations greater than 65%.

The correlation matrix of the set of variables provided in Fig. 2 demonstrates the strong separation between structural and textural variables, with a few noticeable exceptions: coefficient of variation of the histogram (${\mathrm{CoV}}_{\mathrm{HIST}}$) and max. ${\text{probability}}_{\mathrm{GLCM}}$ aligned with $g_{\left[0.95\right]}$ and ${\overset{˜}{g}}_{\left[0.95\right]}$, and ${\text{entropy}}_{\mathrm{GLCM}}$ and ${\text{homogeneity}}_{\mathrm{GLCM}}$ with $H_{0}$.

#### 3.2.2 Principal components analysis

Scaled PCA of the dataset (which is based on the correlation matrix of Fig. 2) provides further insight into how various quantitative features considered capture the information available. For the sarcoma dataset under study, the first 15 principal components explain 95% of the variance, and each further component captured less than 1% of additional information.

#### 3.2.3 PCA-guided clustering

In order to identify variable groupings in the PCA domain that were relevant to the patient information, we performed clustering of the PCA-projected features, using their transformed coordinates in the subspace spanned by the first 15 principal components. Clustering was performed via k-means^33^ of the projected variables into 15 clusters, based on the above considerations. Other strategies may be used to guide variable clustering. Table 1 summarizes the compositions of the resulting variable groupings, which tend to align with distinct functional interpretations. Clusters $C_{1}$ to $C_{15}$ may, respectively, be identified in terms of patient and histologic information ($C_{1}$ to $C_{4}$), assessment of volume and morphology ($C_{5}$ to $C_{6}$), metrics related to metabolic avidity ($C_{7}$), structural heterogeneity ($C_{8}$), assessment of distributional variability and correlation ($C_{9}$ to $C_{11}$), and metrics predominantly related to intensity levels ($C_{12}$ to $C_{15}$).

**Table 1:** Composition of the 15 k-means clusters of the principal components. Remarkably, routine clinical variables grade, subtype, age, and sex each defines their own cluster. Volume and morphologic indicators are grouped into clusters $C_{5}$ and $C_{6}$ together with the (GLSZM) regional features. $C_{9}$ and $C_{10}$ are the only clusters containing both structural and textural features.

Cluster	Variables in cluster
$C_{1}$	Grade
$C_{2}$	Subtype
$C_{3}$	Sex
$C_{4}$	Age
$C_{5}$	${\mathrm{PCA}}_{\mathrm{flatness}}$, ${\mathrm{PCA}}_{\mathrm{elongation}}$
$C_{6}$	Volume, TLG, asphericity, ${\mathrm{PCA}}_{\mathrm{major}}$, ${\mathrm{PCA}}_{\mathrm{minor}}$, ${\mathrm{PCA}}_{\mathrm{least}}$, ${\mathrm{Size}\text{-}\mathrm{zone}}_{\mathrm{GLSZM}}$, ${\mathrm{Intensity}}_{\mathrm{GLSZM}}$
$C_{7}$	SUVmax, SUVmean, $g_{\left[0.25\right]}$, $g_{\left[0.95\right]}$, $g_{Y,[0.25]}$, $g_{Y,[0.95]}$
$C_{8}$	$H_{0}$, $H_{1}$
$C_{9}$	${\tilde{g}}_{\left[0.25\right]}$, ${\mathrm{correlation}}_{\mathrm{GLCM}}$
$C_{10}$	${\tilde{g}}_{\left[0.95\right]}$, ${\mathrm{skewness}}_{\mathrm{HIST}}$, ${\mathrm{kurtosis}}_{\mathrm{HIST}}$, ${\mathrm{CoV}}_{\mathrm{HIST}}$, ${\mathrm{uniformity}}_{\mathrm{HIST}}$, ${\mathrm{uniformity}}_{\mathrm{GLCM}}$, ${\mathrm{homogeneity}}_{\mathrm{GLCM}}$ max. ${\mathrm{probability}}_{\mathrm{GLCM}}$
$C_{11}$	${\mathrm{Variance}}_{\mathrm{HIST}}$, ${\mathrm{entropy}}_{\mathrm{HIST}}$, ${\mathrm{entropy}}_{\mathrm{GLCM}}$, ${\mathrm{dissimilarity}}_{\mathrm{GLCM}}$, ${\mathrm{contrast}}_{\mathrm{GLCM}}$
$C_{12}$	Min. ${\mathrm{gradient}}_{\mathrm{HIST}}$, max. ${\mathrm{gradient}}_{\mathrm{HIST}}$
$C_{13}$	Min. gradient ${\mathrm{gray}}_{\mathrm{HIST}}$
$C_{14}$	Max. gradient ${\text{gray}}_{\mathrm{HIST}}$
$C_{15}$	${\text{Mean}}_{\mathrm{HIST}}$, ${\mathrm{median}}_{\mathrm{HIST}}$, ${\text{autocorrelation}}_{\mathrm{GLCM}}$

### 3.3 Feature Selection

The above feature space exploration indicates that structural and radiomic variables tend to span different areas of the information space. This suggests that (i) structural variables derived from spatial modeling of the volumetric FDG uptake distribution have prognostic potential in the analysis of sarcomas and (ii) they remain useful when used in combination with texture features commonly used in radiomics. The availability of many quantitative variables for the characterization of tumor metabolism raises the question of final selection of prognostic variables. Multivariate prognostic models may be defined directly from available features (for instance, using only structural features, only radiomic features, or a combination of both), or on the basis of a feature selection approach. We considered several typical alternatives for this purpose, exploring two avenues: using principal components analysis and clustering; and using typical machine learning classifiers. These methodologies are described hereafter.

#### 3.3.1 Feature selection guided by PCA and clustering

PCA can be used directly to create multivariate prognostic models, as it organizes the input patient information in an optimal recombination. On the basis of the PCA of Sec. 3.2.2, we considered a prognostic model made of the first 15 principal components.

One downside of a PCA-guided prognostic analysis is that the variables used in the risk model are not as easily interpretable from a clinical viewpoint (each of the PCA-transformed variables being linear combinations of all input variables $\left\{z_{1},\ldots ,z_{p}\right\}$). Another possible strategy for defining a final set of prognostic variables relies on a clustering analysis of the principal components, e.g., via k-means,^33^ so as to define groupings of the original variables $\left\{z_{1},\ldots ,z_{p}\right\}$ based on the proximity of their images $\left\{z_{1^{\prime }},\ldots ,z_{p^{\prime }}\right\}$ in the PCA-transformed domain. From the clustering output of Sec. 3.2.3, which is summarized in Table 1, final feature selection may be carried out arbitrarily. We used the k-means centroids of the PC clusters as risk covariates in the Cox model. Following this approach, the original Cox model

$$\lambda (t;z)=\lambda_{0}(t)e^{\beta_{1}z_{1}+\ldots +\beta_{p}z_{p}}$$

reduces to the linear recombination:

$$\lambda (t;z)=\lambda_{0}(t)e^{{\overset{˜}{\beta }}_{\left(1\right)}(z_{1}^{\left(1\right)}+\ldots +z_{p_{1}}^{\left(1\right)})+\ldots +{\overset{˜}{\beta }}_{\left(C\right)}(z_{1}^{\left(C\right)}+\ldots +z_{p_{C}}^{\left(C\right)})},$$ (8)

where $z_{k}^{\left(c\right)}$ is the k’th of the $p_{c}$ covariates contained in cluster c∈{1,…,C}, C<p. In other words, the parametrization β of the parametric regression part of the Cox model becomes a reparametrization $\tilde{\beta }$ based on the linear combination of input covariates obtained from the clustering. This PCA-guided approach therefore provides a way of grouping risk variables and aggregating the resulting hazard ratios in terms of how the survival information is spanned by these groups of variables. This is not unlike the original PCA output, which provides PCs as linear combinations of the input variables, except that here we would make use of the clustering analysis of these PCs. This increases the potential for interpretability if a particular feature can be identified to summarize each cluster.

Other approaches could be considered to define or elect variables representative of each principal component (or cluster). One possible strategy is to elect in each cluster the covariate yielding the single highest hazard ratio in absolute value and using these k covariates only, discarding all other variables from the clusters. Further exploration of such alternatives will be the topic of future work; the results presented here are rather provided to suggest this alternative route as a possibility for prognostic evaluation.

#### 3.3.2 Feature selection based on machine learning

A final set of prognostic covariates may be selected using machine learning techniques, such as random forests or neural networks. For radiomics-based analyses especially, which require sieving through a number of features that tend to be much larger than the cohort size, various publications^10^^,^^21^ describe results obtained from different machine learning approaches. However, and more generally for PET-based prognostic assessment, there are no specific benchmark studies or consensus as to a more adequate approach in the literature.^21^^,^^34^^–^^36^ Moreover, machine learning classifiers tend to be considered more naturally than regressors for the selection of PET-derived features^21^; for example, in the form of a k-nearest neighbor classifier for patient outcome^11^ or a neural network classifier for therapeutic response.^10^ In the sarcoma dataset, we consider here that patient outcome information is available in the form of overall patient survival (alive or dead) and duration of survival at last follow-up, so assessment of variable importance may be performed both in classification and regression terms. Here, we follow the classification approach. The incorporation of time-to-event data in a feature selection study using, e.g., random survival forests^37^ and other adapted machine learning techniques^38^ will be considered in future work.

##### Classifiers

The statistical methods used include stepwise selection (both forward and backward) for multivariate logistic regression,^39^ simulated annealing based on linear discriminant analysis^40^ (leaps-and-bounds led to comparable results), the LASSO,^41^ random forests,^42^ neural networks,^43^^,^^44^ and support vector machines (SVMs).^41^^,^^45^^,^^46^

##### Settings and tuning

In terms of specific settings, forward-stepwise selection for logistic regression was initialized with grade as baseline. The LASSO regularization parameter was selected via cross-validated misclassification error.^47^ Random forests were tuned for the number of variables randomly sampled as candidates at each split and using 500 trees (choice of the latter did not impact results significantly).^40^ Two neural networks were compared: a first network with one hidden layer of size 10 and another network with two hidden layers of respective sizes 5 and 2. Both neural networks were trained on scaled input data without tuning using five repetitions for training, a nonlinear activation function, and a conservative 0.01 learning rate.^44^ SVMs were applied to scaled input data and using radial kernels and were tuned for regularization cost and kernel smoothing.^48^ In all cases, tuning was achieved on the basis of 10-fold cross-validation.

##### Performance and variable selection rates

A total of M=100, 70% to 30% split-set cross-validation loops were carried out using randomized training sets of 127 subjects and test sets of the remaining 55 subjects. This cross-validation was performed primarily in order to assess and account for the variability in feature selection. The machine learning classifiers were retuned at every cross-validation step for the corresponding training set.^40^^,^^42^^,^^43^^,^^49^

The classification framework was formed on the basis of binary patient outcome. Variable importance and/or variable selection rates were assessed from the training set. For neural networks and random forests, final variable sets were obtained from each algorithm by retaining the top K variables, ranked by decreasing order of importance, successively for all values of K∈{6,…,16} (yielding 11 models for each of these approaches). Variable importance was defined as the Gini index^45^^,^^50^^,^^51^ for random forests and using the Olden metric for neural networks.^52^^,^^53^ Classification rate, ROCs, and AUCs were evaluated from performance on the test sets. With the results from these experiments, presented in the next section, we aim to demonstrate that various selection schemes (i) tend to elect structural variables (including metabolic gradients) and (ii) attempt to combine structural and texture features in the final feature set.

## 4 Results

In the outputs presented below, g, g.n, and g.w, respectively, denote g, $\overset{˜}{g}$, and $g_{Y}$; 0.0.25 and 0.0.95, respectively, the 25th and 95th percentiles; .HIST, .GLCM, and .GLSZM features derived from the histogram, GLCM, and GLSZM, respectively.

### 4.1 Feature Selection from Outcome-Based Classifiers

#### 4.1.1 Outcome-based classification performance

**Fig. 3:**
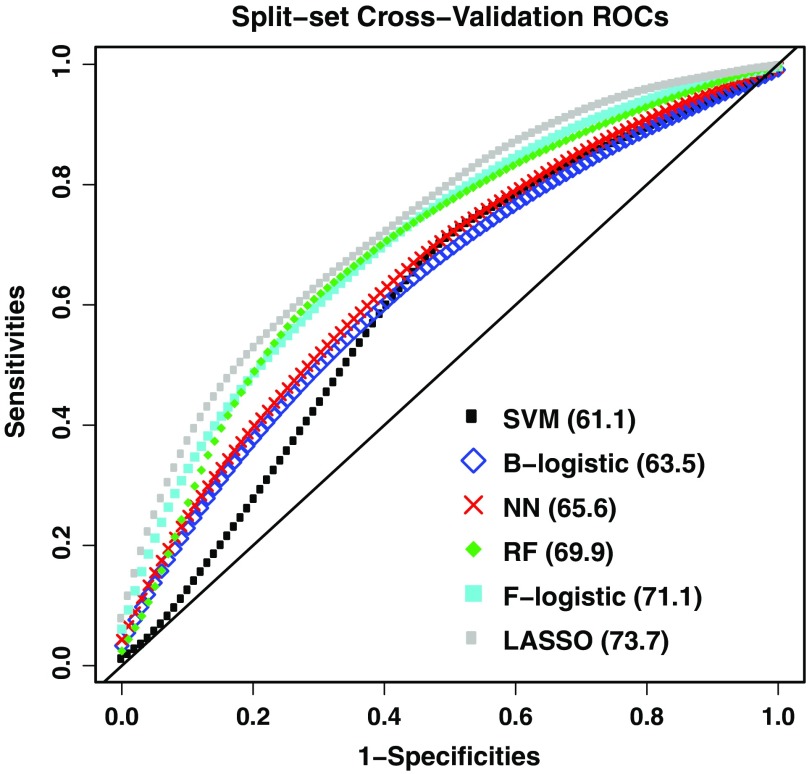
Receiver operating characteristic curves for the classifiers for outcome-guided feature selection (SVM: support vector machines; F/B-logistic: forward- and backward stepwise logistic regression; NN: neural networks; RF: random forest). AUCs are specified in increasing order (inset). Random forests, forward-stepwise logistic regression, and the LASSO yielded effective discrimination rates.

Figure 3 shows the cross-validated ROCs obtained by averaging ROCs across all M=1000 cross-validation samples for all classifiers (except the single-layer neural network, which did not perform better than the two-layer network and was left out of further analyses). The corresponding cross-validated AUCs indicate that the LASSO, forward-stepwise logistic regression, and random forest models yield close, acceptable performance, and outperform the other classifiers. Interestingly, different classifiers tend to select very different feature sets, as we see next.

#### 4.1.2 Feature selection rates per classifier

**Fig. 4:**
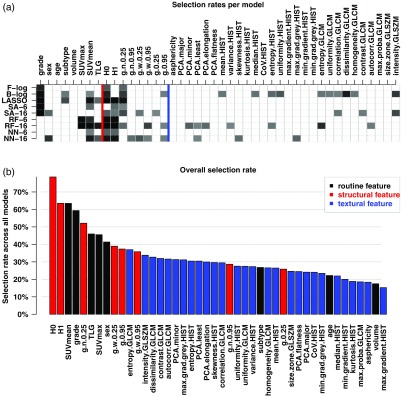
(a) Feature selection rates per classifier. F/B-log: forward- and backward stepwise logistic regression; SA-k: simulated annealing; RF-k: random forest; NN-k: neural networks; where “-k” denotes using the k most important features on average from that model (hence considering 6-variable and 16-variable models). Selection rates below 50% were coded in white; rates above this threshold are represented with a proportionally darker color. The two vertical lines are supplied as visual guides to identify the routine (left-most), structural (central), and textural (right-most) subsets of variables. (b) Feature selection rates overall, i.e., combined across all M=1000 cross-validated experiments, for the same subset of six classifiers comprising of both logistic classifiers, the LASSO, and simulated annealing, random forest and neural network classifiers with k=16. Features are ranked in decreasing order of popularity and color-coded according to feature frame.

The top plot of Fig. 4 summarizes cross-validated variable selection rates for each classifier. This method-specific summary highlights features that were selected for over 50% of the M=1000 cross-validation samples–a darker box indicating a higher selection rate. Rates are shown for “smaller” (k=6) and “larger” (k=16) models in order to emphasize how feature selection evolved with k. Surprisingly, the random forests and the neural networks did not elect grade consistently as one of the most predominant features, unlike the more traditional methods (stepwise logistic regression, LASSO, and simulated annealing). Some structural variables, and in particular, $H_{0}$ and $H_{1}$, are selected rather consistently. Random forests and neural networks noticeably favor a selection of structural features over grade (the former may capture grade-related aspects of the tumor characteristics^2^). For radiomic features, the selection process is less systematic, in that variables are elected either less often or in different combinations for varying cross-validation samples.

Table 2 presents the multivariate prognostic analyses based on the most popular models obtained from five selection schemes: forward-stepwise logistic regression selection, simulated annealing, the LASSO, random forests, and neural networks. For each method, the k=15 most frequent variables were identified. Of these subsets, those variables selected for over 50% of Monte Carlo repetitions were used together into a multivariate model. The table indicates that clinical and structural features are selected consistently by all methods and that at least one gradient summary is among these variables deemed most important by the selection scheme.

**Table 2:** Cox proportional hazard model analyses of multivariate prognostic models comprising of only clinical and structural variables, only clinical and radiomics variables, combining both, or obtained from forward logistic selection, simulated annealing, the LASSO, random forests and neural networks with k=15 variables. In each model, the variables selected by the corresponding technique for over 50% of the M=1000 cross-validation sets were used. This cut-off selection rate was chosen arbitrarily but different values yield to similar conclusions. This table indicates both the most frequently selected features for each model, and contributions that are significant at the 5% significance level (in bold).

	Model [HR (p)]							
Variable	Structural	Radiomics	Combined	F-logistic	SA	LASSO	RF	NN
Grade (int)	**0.46 (0.0020)**	**0.44 (0.0009)**	**0.48 (0.0034)**	−0.47**(0.0029)**	**0.46 (0.0032)**	**0.47 (0.0061)**	—	—
Grade (high)	**0.21 (0.0035)**	**0.20 (0.0022)**	**0.21 (0.0030)**	−0.21**(0.0030)**	**0.19 (0.0022)**	**0.21 (0.0047)**	—	—
Sex (male)	—	—	—	—	1.45 (0.1116)	—	—	1.34 (0.2177)
Subtype (cart)	—	—	—	—	—	0.67 (0.5541)	—	—
Subtype (STS)	—	—	—	—	—	1.14 (0.6119)	—	—
SUVmean	—	—	—	—	—	**1.24 (.0259)**	**1.63 (0.0105)**	1.24 (0.2011)
SUVmax	1.13 (0.2658)	**1.56 (0.0007)**	**1.47 (0.0062)**	—	—	—	0.81 (0.5959)	—
TLG	—	—	—	—	—	0.99 (0.9012)	1.09 (0.6597)	1.00 (0.9806)
$H_{0}$	—	—	—	**1.42 (0.0001)**	1.23 (0.0871)	1.24 (0.0818)	**1.54 (0.0166)**	0.96 (0.8053)
$H_{1}$	**1.89** (${<10}^{-5}$)	—	**1.78 (0.0017)**	—	**1.35 (0.0217)**	**1.36 (0.0139)**	1.07 (0.7337)	**2.32 (0.0004)**
${\overset{˜}{g}}_{\left[0.25\right]}$	—	—	—	**0.83 (0.0438)**	**0.68 (0.0026)**	0.83 (0.0744)	0.93 (0.5722)	—
$g_{Y,[0.25]}$	—	—	—	—	**1.41 (0.0077)**	—	—	1.13 (0.4705)
${\tilde{g}}_{\left[0.95\right]}$	**1.46 (0.0101)**	—	**1.99 (0.0009)**	—	—	—	—	**1.94 (0.0072)**
$g_{Y,[0.95]}$	—	—	—	—	—	—	0.88 (0.6935)	—
${\mathrm{PCA}}_{\text{minor}}$	—	—	—	—	—	—	1.02 (0.9327)	—
${\mathrm{PCA}}_{\mathrm{elongation}}$	—	—	—	—	—	—	0.96 (0.7048)	—
Max. grad.${\mathrm{Gray}}_{\mathrm{HIST}}$	—	—	—	—	—	—	—	1.16 (0.1955)
${\mathrm{CoV}}_{\mathrm{HIST}}$	—	**0.42 (0.0002)**	**0.44 (0.0053)**	—	—	—	—	—
${\mathrm{Skewness}}_{\mathrm{HIST}}$	—	—	—	—	—	—	—	1.00 (0.9961)
${\mathrm{Entropy}}_{\mathrm{HIST}}$	—	**0.03 (0.0003)**	**0.12 (0.0443)**	—	—	—	0.45 (0.2353)	—
${\mathrm{Contrast}}_{\mathrm{GLCM}}$	—	—	—	—	0.85 (0.1851)	—	—	—
${\mathrm{Entropy}}_{\mathrm{GLCM}}$	—	**84.59 (0.0019)**	15.56 (0.0603)	—	—	—	1.83 (0.3316)	—
${\mathrm{Homogeneity}}_{\mathrm{GLCM}}$	—	**6.35 (0.0074)**	2.79 (0.1274)	—	—	—	—	—
${\text{Intensity}}_{\mathrm{GLSZM}}$	—	—	—	—	0.95 (0.6064)	—	—	—
Concordance	0.72	0.72	0.74	0.70	0.73	0.73	0.69	0.70

**Fig. 5:**
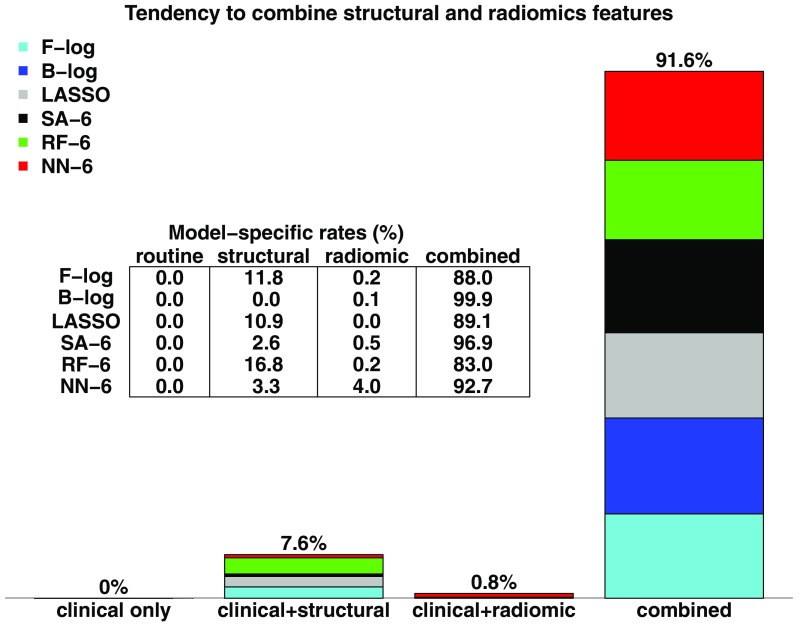
Rates of selection of either clinical-only models, structural-only models, radiomics-only models, or models that combine (at least) structural and radiomic features, out of all cross-validation samples and from the logistic models, the LASSO and six-variable simulated annealing, random forest and neural network classifiers considered.

There was more variability in the selection of textural features (partly due to the textural frame being larger than the other two feature frames). These lower selection rates do not indicate that this quantitation strategy is less effective for prognosis (Fig. 5, described further, indicates the contrary), but rather that several groupings of these features seem to hold comparable prognostic potential. In other words, different subgroups are in turn important for different cross-validation samples of the cohort. This suggests that the information captured by these conventional radiomic features may be more versatile.

#### 4.1.3 Overall feature selection trends

The bottom plot of Fig. 4 summarizes cross-validated variable selection rates aggregated across the following classifiers: the backward- and forward-stepwise logistic models, and the k=16 most important variables elected by simulated annealing, random forests, and neural networks (thus allowing as many features as possible from our framework). Only one type of each classifier was considered here in order to not artificially inflate these selection rates.

This graphic highlights the prevailing importance of the routine clinical variables, as would be expected from experience with sarcoma. It also emphasizes the high impact of structural features $H_{0}$, $H_{1}$, and the first quartiles of normalized metabolic gradients (these three variables were all selected for over 50% of cross-validation samples). This analysis aligns with previous experience^2^^,^^3^ of the two structural heterogeneity measures $H_{0}$ and $H_{1}$, but it also underlines the potential role of metabolic gradient as a additional prognostic variable. The most popular radiomic variables were GLCM and GLSZM features, but many have comparable rates, because different radiomic combinations tend to be used for different cross-validation samples.

Furthermore, our results indicate that most model selection techniques tend to associate both structural and textural features. Figure 5 illustrates that in a majority of cases, classifiers elect models that combine structural and textural features (as well as clinical ones). This figure indicates rates for the two stepwise logistic models, the LASSO, simulated annealing, random forest, and neural network classifiers with a required k=6 variables. It is remarkable that these two families of features tend to be combined even for smaller models. These sets of experimental results show that although the nature of the features finally selected varies greatly with the choice of feature selection technique, models that are deemed optimal most of the times combine both types of PET-based quantitations of tumor characteristics.

### 4.2 Outcomes Analyses

#### 4.2.1 Outcomes analyses from conventional models

Table 2 includes structural-only and radiomics-only prognostic models to explicitly demonstrate the prognostic potential of either feature frame in sarcoma. These two models were selected as they include the same clinical variables, have optimal AIC, and achieve high concordance compared to other structural-only and radiomics-only models. This analysis establishes the significance of texture analysis for prognostic modeling of overall survival (Vallières et al.^20^ showed the predictive potential of texture analysis in a joint PET-MRI predictive model for the presence of lung metastasis in sarcoma). Similarly, structural variables $H_{1}$ and ${\overset{˜}{g}}_{\left[0.95\right]}$ are prognostic when used instead of the radiomic features.

**Fig. 6:**
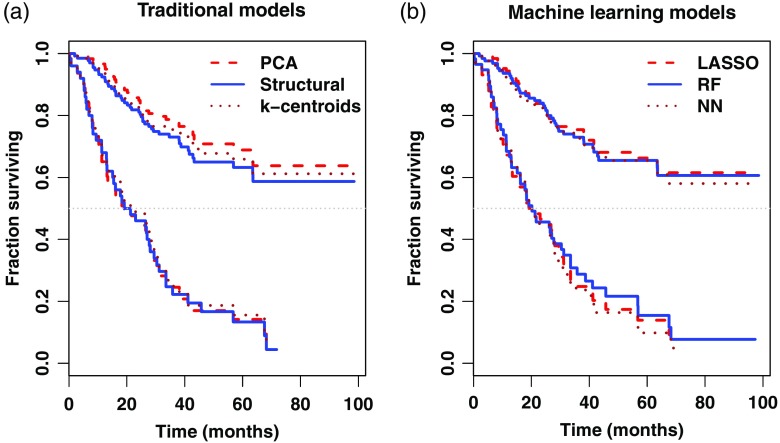
Kaplan–Meier survival curve estimates for dichotomic (low- versus high-) risk group segregation on the basis of various feature selections. For each model, the corresponding two Kaplan–Meier curves were obtained by maximizing the risk group separation in terms of highest log-rank test statistic via grid search. (a) Analyses using models derived from PCA model, k-means centroids Eq. (8), and AIC-based selection of structural variables (out of $H_{0}$, $H_{1}$, and the gradient summaries). (b) Similar Kaplan–Meier analyses for models obtained from machine learning techniques (LASSO, random forests, and neural networks). All models, although different, yield comparable survival analysis performances and significant risk stratification (log-rank test $p<10^{-7}$).

Figure 6 shows Kaplan–Meier analyses obtained from various multivariate risk models. Each analysis stratifies the sarcoma cohort into lower- and higher-risk groups on the basis of maximum log-rank test statistic so as to maximize the dichotomic separation. In all cases, separation was statistically significant ($p<10^{-7}$). The left-hand-side panel shows output from three traditional models: the structural model of Table 2 (solid lines), the prognostic model comprising of the PCA components of Sec. 3.2.2 (dashed lines), and the k-means centroid model Eq. (8), using the 15 cluster centroids as risk predictors (solid lines). Although risk stratification varies with the model, risk separation remains comparable.

In the multivariate Cox proportional hazard analysis for the k-means centroid model Eq. (8) used in Fig. 6, significant cluster centroids were those of clusters $C_{1}$, $C_{7}$, $C_{8}$, and $C_{9}$ ($p<10^{-4}$, p=0.0369, p=0.0129, and p=0.0096, respectively), which are all summaries of routine clinical and structural assessment (cf. Table 1). In this model, the grouping of a relatively small number of structural features captures critical prognostic information for the cohort, unlike the summaries of conventional radiomic features.

#### 4.2.2 Outcome analyses from machine learning classification output

In terms of prognostic validation, multivariate proportional hazard analyses summarized in Table 2 show that at least one structural variable is significant in each of the prognostic models and that structural variables remain significant risk factors in the absence of grade. It also demonstrates that structural assessment of sarcoma tumors is useful in the presence of various combinations of conventional radiomic features. Although no radiomic feature is prognostic in this presentation, some were significant in cross-validation survival analyses.

The right-hand-side panel of Fig. 6 illustrates similar Kaplan–Meier analyses using models obtained from the LASSO (dashed lines), random forests (solid lines), and neural networks (dotted lines) methodologies of Sec. 3.3.2. These curves are provided to illustrate that although the underlying multivariate models may be quite different, they may still capture common characteristics of overall patient risk. The choice of a selection technique used in building a prognostic model may not necessarily be critical in this regard.

## 5 Discussion

### 5.1 Summary of Findings

Building on the results of recent spatial modeling approaches for the analysis of sarcoma,^2^^,^^3^ this paper presents a variable, namely a measure of metabolic gradient, for further characterization of the metabolic activity in sarcoma tumors based on FDG-PET imaging data. A previous uptake profiling approach^2^ has been adapted to allow for a coherent calculation of gradients of the metabolic uptake profile obtained from a spatial model of the tracer uptake distribution. The profile and gradients are evaluated at each voxel and can therefore be mapped onto the imaged volume of interest for visualization. This sample of gradients can also be summarized into a single value (for example, its 95th percentile), to provide opportunities for multivariate prognostic evaluation along with other features derived from the structural representation of the VoI. Here, we used this information in three forms by considering the raw, uptake-normalized, and uptake-weighted gradients. Each of these summaries has its own practical interpretation, but overall they all evaluate local rates of change in glycolysis.

This paper also demonstrates that the proposed model-based structural variables (heterogeneity and metabolic gradients) complement characteristics captured by radiomic features, in that each quantitation methodology captures distinct parts of the information space. Ellipsoidal morphologic features were especially of interest given they are based on the same reference shape as is our model. In previous analyses (e.g., by O’Sullivan et al.^54^), morphology was not found to be an independent risk factor for sarcoma prognosis. Here, we found no correlation between our metrics and those morphologic features, which rather capture volumetric aspects. Similarly, no relationships were found between metabolic gradient summaries and conventional histogram gradient metrics.

Different mechanisms were considered for feature selection, including PCA and machine learning techniques, which are particularly suited to the PET imaging analysis context, where the number of covariates is likely to be (much) greater than the size of the cohort. In total, 37 schemes were considered, including the LASSO, random forests, and neural network models. It was seen that feature selection varied with the technique considered. Overall, structural variables assessing heterogeneity and metabolic gradients were rated among the most important prognostic variables across all methods, along with routine variables, such as tumor grade and SUVmean. The importance of these features may vary with the disease and imaging modality but appears to be relevant in the case of FDG-PET imaging of sarcoma.

In summary, the contribution of this paper is threefold. (i) It defines a promising approach for evaluating the tumor metabolic gradient based on FDG-PET imaging data and validates its statistical significance in prognostic modeling of overall patient survival. Through this process, the paper also demonstrates the (separate) prognostic utility of typical texture features in sarcoma. (ii) It demonstrates that descriptors derived from our spatial model of PET tracer uptake capture aspects of patient information that complement those described by morphologic and textural features. (iii) It illustrates that structural and conventional radiomic features can be successfully combined for multivariate prognostic modeling from a range of statistical learning techniques.

### 5.2 Discussion

Structural variables allow for both quantitative and qualitative assessment due to interpretability of their underlying model and could be used in various ways in clinical settings. For example, image mapping of the metabolic rates could be used to guide understanding of the metabolic process, e.g., in predicting areas in the volume more likely to see an increase in glycolysis at short term. More generally, both structural and textural features are typically used to measure (some form of) intratumoral heterogeneity, and the literature often understands or summarizes these quantitations without explicit distinction in many contributions.^1^^,^^2^^,^^5^^,^^17^ Some descriptions of these approaches acknowledge subtle differences in what they capture, by referring to, e.g., spatial or textural heterogeneity.^1^^,^^2^^,^^5^ It is usually accepted that PET imaging allows one to assess macroscopic heterogeneity, which is not clearly linked to microscopic heterogeneity in the literature. Recent works suggest that only a small number of texture features may actually correlate with microscopic assessment (notably some work on mice^55^), but no report clearly establishes a correlation with other macroscopic assessments. On the other hand, structural analyses, such as the ellipsoidal-based template approach considered here, have links to histologic grading of sarcomas^2^ (spatial modeling of the tracer uptake distribution also has the ability to capture other characteristics of the disease). These two methodologies may in fact capture very different aspects of the tumor histologic profile. Simple examples can highlight that texture features assess tumor characteristics that are not structural in nature; a toy example presented in Appendix B illustrates this.

Incorporation of subtype into the analysis was not found significant in any of the prognostic models present in this work–this is consistent with the result reported by Eary et al.^2^ The observed complementarity between structural and radiomic variables may merit more detailed examination of sarcoma subtypes information. Further exploration of this issue may be warranted.

The structural methodology proposed here is segmentation-insensitive by nature, as technically it does not rely critically on the whole sample of observations. For example, lower extreme values can be removed or down-weigthed for model-fitting without impacting the output analysis. This is not true for textural analyses, where any processing of the input sample will directly affect the GLCM and other critical features that the quantitation may rely on.

The clinical dataset analyzed here is attractive by its size (sarcoma having relatively low prevalence) and the breadth of patient follow-up information. It was, however, acquired using an old-generation tomograph using FBP reconstruction. Many publications have highlighted the significant and varying influence of image reconstruction settings on texture features in 18F-FDG PET^30^^,^^56^^–^^58^ and the question also arises for our model-based quantitation. A simulation study presented in Appendix C illustrates the relative stability of model-based quantitations with respect to the choice of PET reconstruction technique applied to a 2-D simulated phantom. It shows that for both FBP and maximum likelihood (ML) reconstruction techniques, error characteristics for $H_{0}$, $H_{1}$, and ${\overset{˜}{g}}_{\left[0.25\right]}$ are comparable and with decreasing variance as count increases. Our modeling approach was also successfully carried out on a clinical PET/CT nonsmall cell lung dataset acquired between 2012 and 2015 on a GE Discovery VCT with OSEM reconstruction. Exploratory analyses demonstrated a similar correlation structure between our structural variables and a set of textural features comparable to the one used here.^59^^,^^60^

Final feature selection based on machine learning classifiers suggests a less stable role found for radiomic features. These could either be represented significantly or on the contrary, given much lower importance depending on the method used. This also depends on the classification reference, i.e., what patient outcome or disease information is used to guide the selection problem, if no histological information is available. It seems unlikely that a consensus may be reached as to what feature selection method should be used when implementing radiomics analyses. These considerations may again change when larger sets of texture features are included or when censored time-to-event data is taken into account;^38^^,^^61^ this will be the scope of future investigations.

## Figures and Tables

**Fig. 7:**
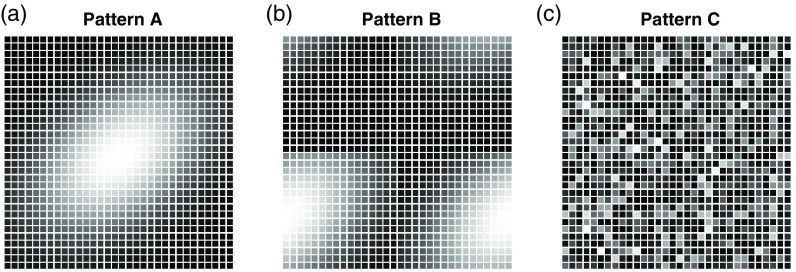
Toy patterns (mid-volume transverse slices). (a) Pattern A, (b) pattern B, and (c) pattern C.

**Fig. 8:**
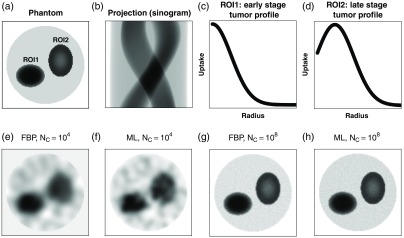
(a)–(d) 2-D phantom data and (e)–(h) reconstruction examples, using filtered backprojection (FBP) and maximum likelihood (ML) at low ($N_{C}=10^{4}$) and high ($N_{C}=10^{8}$) count levels. Note that reconstructions are set to zero in background (defined as the region with no attenuation).

**Fig. 9:**
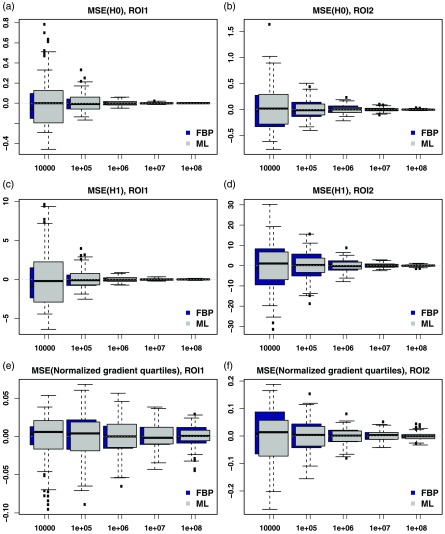
Monte Carlo MSE’s for count levels $N_{C}$ varying in $\left\{10^{4},10^{5},10^{6},10^{7},10^{8}\right\}$ for $H_{0}$ (top), $H_{1}$ (center), and first quartiles of normalized metabolic gradients Eq. (6) (bottom), for both (a) ROI1 and (b) ROI2.

**Fig. 10:**
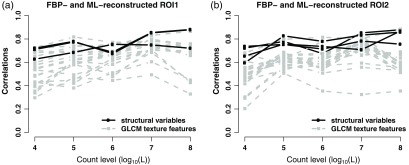
Correlations between Monte Carlo feature estimates obtained from FBP- and ML-reconstructed (a) ROI1 and (b) ROI2, for count levels $N_{C}$ varying in $\left\{10^{4},10^{5},10^{6},10^{7},10^{8}\right\}$. Correlations for structural features $H_{0}$, $H_{1}$, and ${\tilde{g}}_{\left[0.25\right]}$ [Eq. (6)] are shown in black solid lines; those for GLCM features in gray, dashed lines.

## Appendix A: Description of Algorithm

1. Initial ellipsoid location and orientation are calculated using weighted mean and covariance matrix $\left(\mu^{0},\Sigma^{0}\right)$ of $\left\{X_{i}\right\}$. At each iteration, ellipsoidal coordinates are updated via spectral decomposition:
  $$\Sigma =\Gamma D\Gamma^{T},\;D=\Lambda^{-\frac{1}{2}},\;x\mapsto x^{⋆}=\Gamma D^{T}(x-\mu )$$
  to generate ellipsoidal coordinates for voxels i=1,…,n, in the form:
  $$u_{i}(\theta )={\left(x_{i}^{⋆}-\mu \right)}^{T}\Sigma^{-1}(x_{i}^{⋆}-\mu ),$$
  where θ=(μ,Σ). A monotonically decreasing uptake profile is obtained nonparametrically from mean isotonic regression^62^ for heterogeneity evaluation.^2^
2. For bitonic regression, a range of mode values ${\left\{\tau_{m}\right\}}_{m=1}^{M}$ are considered. Given any mode point, the best unimodal fit is found by solving a quadratic programming problem $\underset{i}{\sum }w_{i}{\left[y_{i}-f(u_{i}|\tau_{m})\right]}^{2}$, where f is piecewise constant over a defined set of knots. The knots are set at percentiles of the u-distribution. The mode yielding optimal fit is used. One could project the final fit onto a set of B-splines, if desired, although this is optional.
3. Using a final regular unimodal curve, e.g., using B-splines, is preferable in order to compute metabolic uptake profile gradients for each voxel. Gradients can be evaluated, e.g., using central divided differences from either of the iso- and bitonic regression curves derived above.
  An open-source implementation of this approach in R^39^ is available at https://github.com/ericwol/mia.

## Appendix B: Toy Example

This toy example demonstrates the inability of some popular textural features in capturing structural changes in the volumetric uptake pattern. Here, a 3-D ellipsoidal structure (A) and is spatially rearranged to form two alternative patterns B and C, as shown in Fig. 7. The histograms of the three volumes being identical, all first-order texture features are equal for the three patterns since they are all based on the histogram (cf. Table 3). Overall, if all the second-order features considered in this example do vary significantly between patterns A and C (although second-order entropy by only 20%), some of them, including maximum probability, entropy, uniformity, correlation, and homogeneity, incur only minor variations between patterns A and B, which is a three-dimensional rearrangement of quadrants of the volume of pattern A.

**Table 3:** Percentage-changes in quantitation from pattern A for texture features, illustrating that first-order features are spatially insensitive. Some second-order (GLCM) features also show a lack of sensitivity to drastic structural changes in the spatial distribution of image intensities such as between patterns A and B. It is also notable that changes in contrast have different signs whether we consider pattern B or C.

Percentage-change in quantitation from pattern A					
First-order feature	Pattern B	Pattern C	Second order feature	Pattern B	Pattern C
Entropy	0%	0%	Max. probability	0.4%	−83.4%
Energy	0%	0%	Entropy	0.5%	21.1%
Mean	0%	0%	Uniformity	−0.8%	−75.8%
Variance	0%	0%	Correlation	−2.0%	−99.5%
Skewness	0%	0%	Homogeneity	−3.0%	−55.2%
Kurtosis	0%	0%	Dissimilarity	9.1%	352.8%
Roughness	0%	0%	Autocorrelation	−17.7%	−44.0%
CoV	0%	0%	Contrast	18.1%	−1683.7%

## Appendix C: Effect of Reconstruction

A numerical study was used to explore how heterogeneity and metabolic gradient quantitation derived from our analysis approach varied with dose and the type of reconstruction process used—FBP or ML. A simplified (128×128) 2-D circular tissue phantom including ellipsoidal tumor structures with early (ROI1) and late (ROI2) stage profiles was considered. Uniform attenuation within the circular FoV of the tissue is assumed. The projection domain was discretized uniformly over distance (183 values) and angle (181 values). For a given expected count $N_{C}$, proportional to the injected dose, Poisson data were simulated and subsequently reconstructed using both FBP and full ML (1000 EM iterations starting from the true). Raw reconstructions were filtered (smoothed) using a Gaussian kernel whose bandwidth was optimized to minimize the mean square of the reconstruction error over the circular FoV, where attenuation is nonzero. Expected counts ranged over five levels $N_{C}\in \{10^{4},10^{5},10^{6},10^{7},10^{8}\}$. Sample reconstructions are shown in Fig. 8.

In order to obtain a realistic understanding of statistical variation, 100 Monte Carlo replicates were obtained at each count. Thus, a total of 500 FBP and ML reconstructed images were generated for analysis. RoI data for both tumor regions from each reconstruction were analyzed to extract structural (ellipsoidal modelling) variables. The variables were compared to the average over the replicate values at the highest count data–this is regarded as the true. The observed deviations between individual data estimates and the true are summarized by boxplots in Fig. 9. Error characteristics are dominated by variability; bias plays a negligible role. For both FBP and ML, variance is seen to diminish with count–this indicates that the structural variables become more reliable with increasing count–which is in substantial agreement with an asymptotic analysis of heterogeneity provided by O’Sullivan et al.^54^ At the lowest count, there is an indication that FBP-based structural variables of ROI2 are more variable than ML-based ones; but this is not the cased for ROI1. The simulation clearly shows whatever differences there may be between ML- and FBP-based structural variables, they diminish with count.

We also evaluated texture variables for the simulated FBP and ML data. A full analysis of these data is beyond the scope of this work. Figure 10 shows the relationship (measured by correlations over the replicate samples) between variables obtained from the FBP and ML data using both structural and texture variables. As indicated by previous studies,^30^^,^^56^^–^^58^ a number of texture features shows poor alignment between the two types of reconstructions. The data show that the structural variables are less sensitive to the reconstruction methodology used. A more complete treatment of the issues raised here would be of interest. In future work, we plan to report on this in a 3-D setting.
